# Integrated Analyses Reveal the Multi-Omics and Prognostic Characteristics of ATP5B in Breast Cancer

**DOI:** 10.3389/fgene.2021.652474

**Published:** 2021-05-28

**Authors:** Min Liu, Yuxuan Xu, Yaoyao Zhou, Ronggang Lang, Zhenyu Shi, Jing Zhao, Yuanyuan Meng, Li Bao

**Affiliations:** ^1^Key Laboratory of Cancer Prevention and Therapy, Key Laboratory of Breast Cancer Prevention and Therapy, Tianjin Medical University Cancer Institute and Hospital, National Clinical Research Center for Cancer, Tianjin’s Clinical Research Center for Cancer, Tianjin, China; ^2^The Graduate School, Tianjin Medical University, Tianjin, China; ^3^Department of Breast Cancer Pathology and Research Laboratory, Key Laboratory of Cancer Prevention and Therapy, Tianjin Medical University Cancer Institute and Hospital, National Clinical Research Center for Cancer, Tianjin’s Clinical Research Center for Cancer, Tianjin, China

**Keywords:** breast cancer, ATP5B, prognosis, copy number variation, methylation, immunohistochemical staining

## Abstract

The beta subunit of F1Fo-ATP synthase (ATP5B) has been demonstrated to play an essential role in tumor progression and metastasis. However, there has been no comprehensive pan-cancer multi-omics analysis of ATP5B, while the clinical relevance of ATP5B and its potential mechanism in regulating breast cancer are still poorly understood. In this study, we demonstrated that ATP5B has a higher frequency of amplification than deletion in most cancer types, and the copy number variation (CNV) of ATP5B was significantly positively correlated with its mRNA expression level. DNA methylation analysis across pan-cancer also revealed a strong correlation between ATP5B expression and epigenetic changes. We identified 6 significant methylation sites involved in the regulation of ATP5B expression. Tissue microarrays (TMA) from 129 breast cancer samples, integrated with multiple additional breast cancer dataset, were used to evaluate the ATP5B expression and its correlation with prognosis. Higher levels of ATP5B expression were consistently associated with a worse OS in all datasets, and Cox regression analysis suggested that ATP5B expression was an independent prognostic factor. Gene enrichment analysis indicated that the gene signatures of DNA damage recognition, the E-cadherin nascent pathway and the PLK1 pathway were enriched in ATP5B-high patients. Moreover, somatic mutation analysis showed that a significant different mutation frequency of CDH1 and ADAMTSL3 could be observed between the ATP5B-high and ATP5B-low groups. In conclusion, this study reveals novel significance regarding the genetic characteristics and clinical value of ATP5B highlighted in predicting the outcome of breast cancer patients.

## Introduction

Multiple intra- and intertumor genomic and phenotypic variations, also named heterogeneity, is one of the main characteristics of malignant tumors (Tellez-Gabriel et al., 2016). Heterogeneity of tumors results in differences in occurrence, development, invasion and metastasis ability as well as in the prognosis. Breast cancer is the second most common cause of cancer-related death among women after lung cancer (Sung et al., 2021). Breast cancer has a high degree of heterogeneity and can be classified into four molecular subtypes using PAM50 gene signatures, including luminal A, luminal B, HER2-enriched, and basal-like breast cancer (Parker et al., 2009; Coates et al., 2015). Although great progresses have been made in the diagnosis and treatment of breast cancer, further research is needed due to its high morbidity and mortality rates (Harbeck and Gnant, 2017).

The beta subunit of F1Fo-ATP synthase (ATP5B) is exclusively located on the inner mitochondrial membrane and it is a rate-limiting enzyme that produces cellular ATP through oxidative phosphorylation (von Ballmoos et al., 2008; Xu et al., 2009). Recent researches have shown that ATP5B and the other components of the ATP synthase complex, which are also known as ectopic ATP synthase, are expressed on the plasma membrane of certain type of tumor and normal cells, including non-small cell lung cancer (NSCLC) cells, breast cancer cells, endothelial cells, and others (Chi and Pizzo, 2006; Lu et al., 2009; Xu and Li, 2016; Chung et al., 2020). Several studies showed that ecto-ATP synthase was associated with the superinvasive phenotype, suggesting that its activity may support the progression of cancer, tumorigenicity and metastasis. In prostate cancer, researchers identified that ATP5B expression is predictive of poor overall survival and metastasis-free survival. ATP5B also has a selective tumor cell killing function after binding to an RNA aptamer (Li W. et al., 2017). The latest study examining the mechanisms of drug resistance has found that ATP synthase, especially ATP5B, has a high expression level in HER2-positive breast cancer (Gale et al., 2020). However, interference with ATP5B activity could be an approach to overcome acquired resistance to HER2-targeted therapies and to improve the patient’s prognosis. These results demonstrated that ATP5B has important prognostic significance in different tumors and it may be a new therapeutic target.

In this study, from the perspective of multi-omics, including copy number variations (CNV), mutations, mRNA expression levels, and epigenetic modifications, the genetic characteristics of ATP5B were explored using the pan-cancer datasets from The Cancer Genome Atlas (TCGA). Furthermore, we identified ATP5B as a prognostic marker in a cohort of breast cancer clinical samples and another three public databases, providing a new strategy for breast cancer management.

## Materials and Methods

### RNA-Seq Gene Expression Analysis

TCGA normal and primary tumor RNA-seq datasets were downloaded from UCSC Xena^1^ for pan-cancer analysis of ATP5B mRNA ex-pression. Raw counts of gene expression were converted to transcripts per million (TPM) for further analysis (Rooney et al., 2015). Statistical ranking for ATP5B expression was defined as ATP5B-high and ATP5B-low groups by the top and bottom quartiles, respectively. To identify the differentially expressed genes between the ATP5B subgroups, cut-off values were set at FDR < 0.05 and an absolute fold change larger than 1.5. Gene Ontology (GO) analysis of the significantly differentially expressed genes was performed to examine their molecular functions (MFs) (The Gene Ontology, 2019). Based on the MsigDB database, Gene set enrichment analysis (GSEA) was performed to reveal the biological signaling pathways (Subramanian et al., 2007; Liberzon et al., 2015). Statistical significance was determined by the normalized enrichment score (NES) and the false discovery rate (FDR).

### Mutation and Copy Number Variation Analysis

Copy number variation (CNV) in pan-cancer was defined by running GISTIC 2.0. To extract the high confidence CNVs, the threshold of 0.3 in the Gistic2 copy number value was used for amplifications, −0.3 for deletions, and the values between 0.3 and −0.3 were considered as non-CNVs (Mermel et al., 2011). Significantly mutated genes (SMGs) were determined by using the R package “maftools” in different subgroups of breast cancer (ATP5B-high vs. ATP5B-low). The threshold of FDR < 0.05 was considered significant.

### TMA Cohorts

The expression of ATP5B was analyzed using a tissue microarray (TMA) obtained from Shanghai Outdo Biotech Company (China) (Li H. et al., 2017). The TMA contains 129 primary breast cancer tissues and 71 adjacent normal tissues. All patients underwent initial surgical resection and did not receive prior endocrine therapy, chemotherapy, or radiotherapy. Specimens were handled anonymously according to the ethical and legal standards. The clinicopathological features of all patients are described in Supplementary Table 2. This study was approved by the Ethics Committee of Tianjin Medical University Cancer Institute and Hospital.

### Immunohistochemical (IHC) Staining

The TMA sections (4 μm in thickness) were formalin-fixed and paraffin-embedded. Before staining, the slides were deparaffinized and rehydrated. Then, they were treated with 0.3% hydrogen peroxide to block endogenous peroxidases activity and for antigen retrieval. The sections were incubated with a rabbit anti-ATP5B antibody (Proteintech, 17247-1-AP, 1:200 dilution) overnight at 4°C. After staining with a secondary antibody for 30 min at room temperature, the ATP5B expression was visualized by horseradish peroxidase-diaminobenzidine (HRP-DAB) immunostaining (Issac et al., 2019). The images were viewed and captured using a ZEISS light microscope. The slides were independently reviewed by two pathologists, who were blinded to the clinical parameters. ATP5B protein expression was evaluated according to the staining intensity and the proportion of positive cells, scored with a modified histological score (H-score), where the maximum score is 300 (Taylor et al., 2017).

### Survival Analysis

Not only TMA clinical samples but also the breast cancer data from the TCGA and METABRIC databases^2^ were used to estimate the prognostic function. The Kaplan-Meier method was utilized to evaluate the level of ATP5B expression in association with the overall survival (OS) of the patients (Saluja et al., 2019). We used the R package “survival” and “survminer” to perform survival analysis and corresponding visualization, respectively. In addition, an integrative online database PrognoScan^3^ was used for investigating the prognostic role of ATP5B mRNA for distinct endpoints (Mizuno et al., 2009). The threshold was adjusted to a Cox *p*-value < 0.05.

### Statistical Analysis

Statistical analysis was performed using R version 3.6.3, IBM SPSS for Windows version 21.0 and GraphPad Prism version 8.0 (Yu et al., 2012). The Wilcoxon test was used to examine the significant differences in DNA methylation and ATP5B expression (Stefansson et al., 2015). The correlation of ATP5B mRNA expression with CNV as well as with the DNA methylation level was evaluated by the Pearson correlation coefficient. The Chi-squared test was used to verify the significance of the relationship of ATP5B expression with the clinicopathological features of breast cancer. Survival analysis of the ATP5B expression level was evaluated by the Kaplan-Meier method, and the curves were compared with the log-rank test. Univariate and multivariate Cox regression models were employed to determine whether ATP5B expression was an independent prognostic factor in patients with breast cancer. *p*-values less than 0.05 were regarded as statistically significant. ^∗^, ^∗∗^, ^∗∗∗^, and ^****^ indicate *p*-value < 0.05, 0.01, 0.001, and 0.0001, respectively.

## Results

### CNV and the mRNA Expression Profile of ATP5B in Pan-Cancer

**FIGURE 1:**
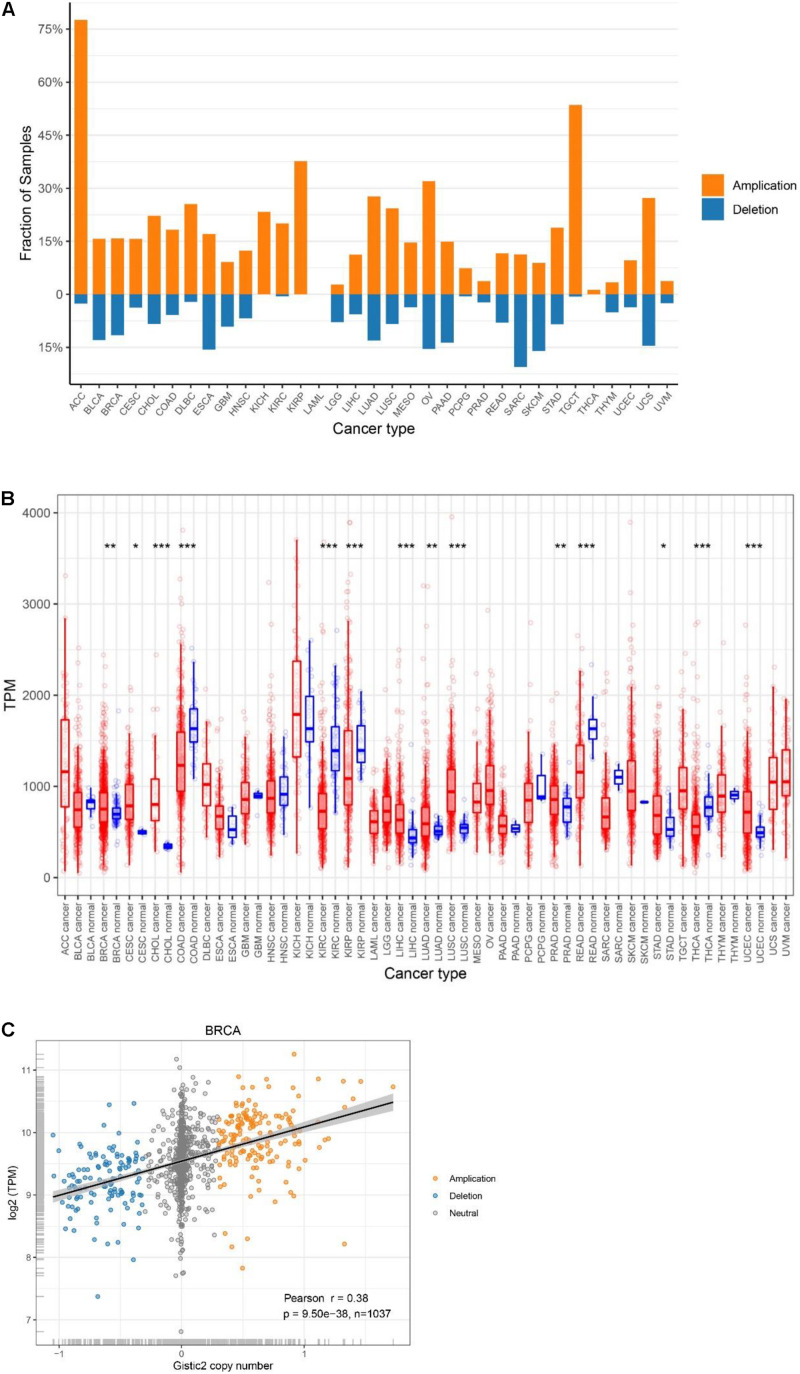
Genetic characteristics of ATP5B in TCGA pan-cancer. **(A)** ATP5B amplification and deletion frequency in TCGA cancer types. ACC, adrenocortical carcinoma; BLCA, bladder urothelial carcinoma; BRCA, breast invasive carcinoma; CESC, cervical squamous cell carcinoma and endocervical adenocarcinoma; CHOL, cholangiocarcinoma; COAD, colon adenocarcinoma; DLBC, lymphoid neoplasm diffuse large B-cell lymphoma; ESCA, esophageal carcinoma; GBM, glioblastoma multiforme; HNSC, head and neck squamous cell carcinoma; KICH, kidney chromophobe; KIRC, kidney renal clear cell carcinoma; KIRP, kidney renal papillary cell carcinoma; LAML, acute myeloid leukemia; LGG, brain lower grade glioma; LIHC, liver hepatocellular carcinoma; LUAD, lung adenocarcinoma; LUSC, lung squamous cell carcinoma; MESO, mesothelioma; OV, ovarian serous cystadenocarcinoma; PAAD, pancreatic adenocarcinoma; PCPG, pheochromocytoma and paraganglioma; PRAD, prostate adenocarcinoma; READ, rectum adenocarcinoma; SARC, sarcoma; SKCM, skin cutaneous melanoma; STAD, stomach adenocarcinoma; TGCT, testicular germ cell tumors; THCA, thyroid carcinoma; THYM, thymoma; UCEC, uterine corpus endometrial carcinoma; UCS, uterine carcinosarcoma; UVM, uveal melanoma. **(B)** Pan-cancer expression landscape of ATP5B. The expression abundance is measured by transcripts per million (TPM). **(C)** ATP5B mRNA expression compared to ATP5B copy number values in breast cancer.

We utilized data generated from 11,057 TCGA pan-cancer samples and analyzed the CNV and mRNA expression levels of ATP5B among all 33 cancer types. Using GISTIC2.0, we found that ATP5B had both amplifications and deletions in 29 cancer types, while there was amplification only in KICH, KIRP and THCA (Figure 1A). ATP5B had a higher frequency of amplifications than deletions in 24 cancer types, including breast cancer, and it was most frequently amplified (>75%) in adrenocortical carcinoma (ACC).

Then, to provide a comprehensive evaluation of ATP5B expression in cancers, we compared ATP5B mRNA expression levels across the pan-cancer datasets, and found diverse expression features of ATP5B (Figure 1B). Among the 24 cancer types which have paired para-cancer tissue data, ATP5B mRNA expression was significantly higher in cancer tissues of 9 cancer types and lower in cancer tissues of 5 cancer types, compared with that of the adjacent normal tissues.

### Analysis of the Potential Genomic and Epigenetic Changes Related to ATP5B Expression

To determine whether ATP5B copy number variations affect ATP5B expression, we examined the correlation between ATP5B CNV and mRNA expression. Among all 33 cancer types, a significant positive correlation was found between ATP5B CNV and the expression level in 28 cancer types (only no correlations for ACC, CHOL, KICH, KIRC, and TGCT) (Supplementary Table 1), including the breast cancer (*r* = 0.38, *p* = 9.5e-38) (Figure 1C). These results suggest that ATP5B is amplified in a significant proportion of cancers and affects mRNA expression to some extent.

**FIGURE 2:**
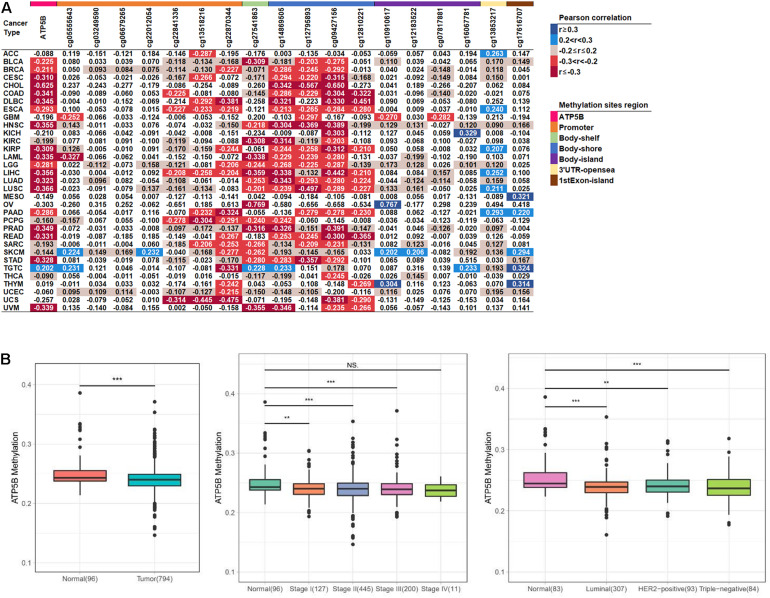
The methylation level analysis of ATP5B in the TCGA datasets. **(A)** Correlation between methylation levels and mRNA expression of ATP5B and its 18 methylation sites across 33 TCGA cancer types. The number represents the Pearson correlation *r*-value, and all statistically significant *r*-values are graded and colored (right legend, *p* < 0.05). **(B)** The ATP5B methylation level in the TCGA breast cancer dataset compared with normal tissues, stages I–IV and the three molecular subtypes. Boxplots represent the median with the interquartile range.

DNA methylation is considered to be an important epigenetic modification as well as one of the main regulators of gene expression (Zhang et al., 2018). Here, we performed an integral pan-cancer analysis of the correlation between ATP5B mRNA expression and DNA methylation, including ATP5B overall methylation level and all 18 methylation sites of ATP5B (Figure 2A). The methylation of ATP5B is negatively correlated with its expression level in 24 cancer types, while only has a positive correlation with the mRNA expression in TGTC (*p* < 0.05). In addition, we identified 6 methylation sites (cg22870344, cg27541863, cg14869505, cg12795893, cg09427156, and cg12810221) that associated with the mRNA expression in more than 10 cancer types, one of which (cg22870344) located in the promoter region. These sites are regarded as significant sites of ATP5B that may be involved in the regulation of expression. In breast cancer, a total of 12 sites were related to its mRNA expression level, including 5 of 6 significant sites described above.

We then evaluated the levels of methylation between the tumor and normal tissues from breast cancer patients. The ATP5B methylation level in tumors was significantly lower than in the normal tissues, which is consistent with the previous results (Figure 2B). Similarly, the ATP5B methylation level was significantly reduced in stage I–III and in all three molecular subtypes (Figure 2B). These results suggest that the reduced ATP5B methylation level in breast cancer increases its expression level.

### Functional Enrichment Analysis of ATP5B Expression Level in Breast Cancer

**FIGURE 3:**
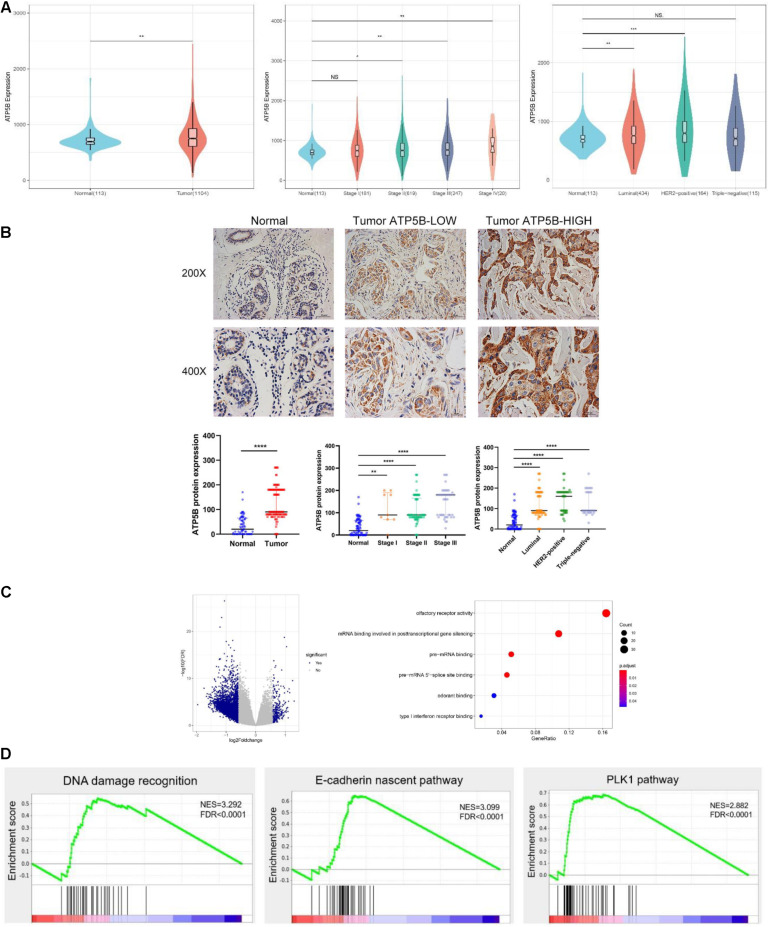
ATP5B expression and functional enrichment in breast cancer. **(A)** Comparison of ATP5B mRNA expression between tumors and normal tissues, stages I–IV, and each subtype, including luminal, HER2 positive, and triple-negative cancers, in the TCGA BRCA data. **(B)** Representative IHC staining of the control, low and high expression of ATP5B protein in breast cancer; scale bar, 20 μm. The relative protein level of ATP5B in breast cancer tissues compared with adjacent normal tissue, stages I–III, as well as the three molecular subtypes. The data represent the median with interquartile range. **(C)** Volcano plot of differentially expression gene profiles between the ATP5B-high and ATP5B-low groups. The cut-off points were set at |log2(fold change)| ≥ 1.5 and FDR < 0.05. GO annotation analysis of the overlapping genes in molecular function. Each circle represents a term, and its size represents the counts of the involved genes. **(D)** The three enrichment plots from the GSEA, including DNA damage recognition, E-cadherin nascent pathway and the PLK1 pathway.

Next, we further analyzed the expression diversity of ATP5B in breast cancer using TCGA BRCA dataset, and the results showed that ATP5B expression was significantly up-regulated in tumors of stages II–IV compared with normal tissues (Figure 3A), and the expression in stage IV was the highest. Consistently, ATP5B mRNA was up-regulated in the luminal and HER2-positive subtypes, while no significant difference was detected in the triple-negative subtype (Figure 3A).

To investigate the clinical relevance of ATP5B in breast cancer, the protein expression of ATP5B was evaluated in a TMA containing 129 breast cancer samples and 71 adjacent normal tissues (Figure 3B). Basically consistent with the results of the mRNA level, the IHC results demonstrated significantly higher protein expression of ATP5B in clinical stages I–III and all three molecular subtypes compared with that in the normal tissues (Figure 3B).

In order to elucidate the functional role of ATP5B in breast cancer, we performed transcriptome analysis based on the TCGA BRCA dataset to compare the gene expression profiles of the ATP5B-high and -low groups. A total of 264 up-regulated genes and 4,746 down-regulated genes were identified in the ATP5B-high group (fold-change ≥ 1.5, using the ATP5B-low group as the reference) (Figure 3C). GO analysis revealed the molecular functions in gene sets such as mRNA binding involved in posttranscriptional gene silencing (Figure 3C).

To evaluate the biologic pathways associated with breast cancer pathogenesis stratified by the ATP5B expression level, we next performed GSEA analysis, which indicated that the gene signatures of the DNA damage recognition, the E-cadherin nascent pathway and the PLK1 pathway were enriched in ATP5B-high patients (Figure 3D). All of these signaling pathways are strongly associated with tumorigenesis and progression (Cai et al., 2016; Li et al., 2019; Biswas, 2020). In conclusion, these results support the potential function of ATP5B as a tumor promoter in breast cancer.

### High ATP5B Expression Impacts the Survival Outcomes of Breast Cancer

**FIGURE 4:**
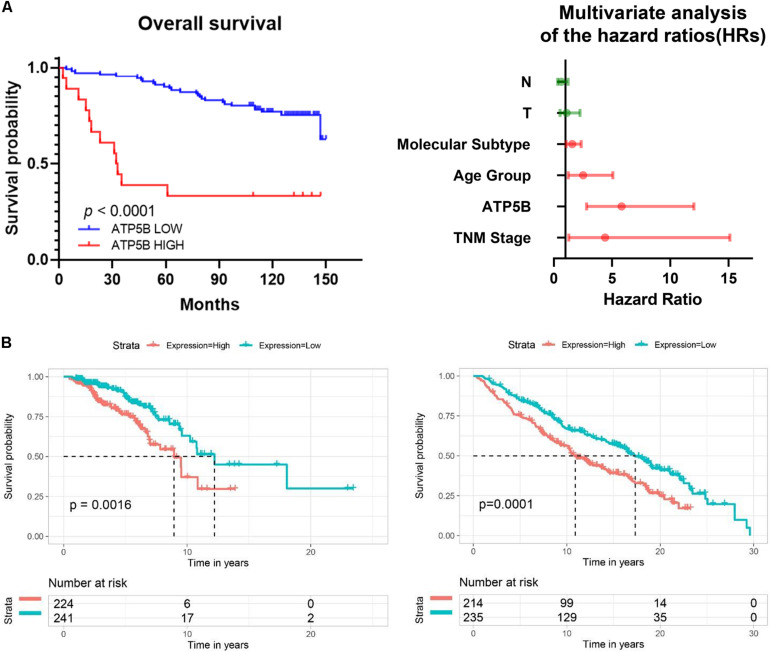
Survival analysis of ATP5B based on clinical samples and online databases. **(A)** Kaplan-Meier (KM) survival curve comparing patients with high and low expression of ATP5B in TMA cohorts. Forest plot of multivariable Cox regression analysis shows that ATP5B is an independent risk factor for OS. Independent prognostic factors, including ATP5B expression and other clinical parameters, were evaluated using both univariate and multivariate Cox proportional hazards analyses. The HRs are presented as the median with 95% confidence interval. Red indicates the significant differences (*p* < 0.05). **(B)** Overall survival of ATP5B based on the TCGA (total 1096 breast cancer samples, left) and the METABRIC (total 1904 breast cancer samples, right) databases.

We used X-Tile software (Camp et al., 2004) to generate the best cutoff point to classify patients of our cohort into subsets with diverse survival outcomes based on ATP5B expression, and we were able to stratify patients into ATP5B-HIGH (*n* = 18) and ATP5B-LOW (*n* = 111) subgroups. The ATP5B-HIGH group had a significantly shorter OS time (*p* < 0.0001; Figure 4A). The two subgroups of different protein expression levels were further correlated with the clinicopathological characteristics of breast cancer. As shown in Table 1, the level of expression of ATP5B was correlated with the lymph node status (*p* = 0.013) and the TNM stage (*p* = 0.023) of breast cancer patients. On the other hand, both univariate and multivariate Cox regression analysis (Shen et al., 2020) showed that ATP5B expression was an independent risk factor for overall survival (Figure 4A and Table 2). The survival results were then validated by using three another independent databases. Consistently, patients with higher ATP5B expression in the TCGA and METABRIC datasets also had worse overall survival outcomes (Figure 4B) and four cohorts (GSE1456-GPL96, GSE3494-GPL96, GSE4922-GPL96, and GSE7390) by using PrognoScan, including different endpoints for breast cancer patients, showed that higher ATP5B expression was associated with an unfavorable prognosis (Table 3).

**TABLE 1:** Association between ATP5B protein expression and the clinicopathological characteristics of breast cancer patients.

Characteristics	ATP5B protein expression				*p*-value
	
	High		Low		
Age group					0.954
<65 years	14	13.9%	87	86.1%	
≥65 years	4	14.3%	24	85.7%	
Tumor size status					0.575
T1	4	14.8%	23	85.2%	
T2	11	12.4%	78	87.6%	
T3	3	23.1%	10	76.9%	
Lymph nodes status					**0.013^a^**
N0	5	11.1%	40	88.9%	
N1	3	7.3%	38	92.7%	
N2	6	17.1%	29	82.9%	
N3	4	50.0%	4	50.0%	
TNM stages					**0.023^a^**
I	2	22.2%	7	77.8%	
II	5	6.8%	69	93.2%	
III	11	23.9%	35	76.1%	
ER status					0.867
Negative	8	14.5%	47	85.5%	
Positive	10	13.5%	64	86.5%	
PR status					0.131
Negative	13	18.1%	59	81.9%	
Positive	5	8.8%	52	91.2%	
HER2 status					0.807
Negative	13	14.4%	77	85.6%	
Positive	5	12.8%	34	87.2%	
Molecular subtypes					0.484
Luminal	7	11.5%	54	88.5%	
HER2-positive	5	12.8%	34	87.2%	
Triple-negative	6	20.7%	23	79.3%	

*^*a*^Using chi-square test; p < 0.05, statistically significant.*

**TABLE 2:** Uni- and multivariate analysis of covariates of OS.

Covariates	Univariate analysis			Multivariate analysis		
		
	Beta	HR (95% CI)	*p*	Beta	HR (95% CI)	*p*
ATP5B	1.612	5.012 (2.515–9.986)	**<0.0001^a^**	1.761	5.819 (2.818–12.015)	**<0.0001^a^**
Age group	0.696	2.005 (1.028–3.908)	**0.041^a^**	0.921	2.512 (1.246–5.063)	**0.01^a^**
T	0.494	1.639 (0.889–3.023)	0.113	0.091	1.096 (0.534–2.249)	0.803
N	0.347	1.414 (1.021–1.959)	**0.037^a^**	−0.444	0.641 (0.334–1.231)	0.182
TNM stage	0.845	2.328 (1.310–4.137)	**0.004^a^**	1.482	4.403 (1.284–15.102)	**0.018^a^**
Molecular subtype	0.453	1.572 (1.072–2.305)	**0.020^a^**	0.454	1.575 (1.056–2.347)	**0.026^a^**

*^*a*^p < 0.05, statistically significant.*

**TABLE 3:** Survival analysis of ATP5B mRNA in breast cancer patients based on PrognoScan.

Dataset	Endpoint	Number	ln(HR-high/HR-low)	Cox *p*-value	ln(HR)	HR (95% CI-low CI-up)
GSE1456-GPL96	Overall survival	159	0.94	0.012584	1.31	3.70 (1.32–10.35)
GSE1456-GPL96	Relapse free survival	159	1.25	0.000966	1.73	5.62 (2.02–15.66)
GSE1456-GPL96	Disease specific survival	159	1.38	0.001393	1.99	7.29 (2.16–24.63)
GSE3494-GPL96	Disease specific survival	236	0.85	0.015937	1.06	2.87 (1.22–6.79)
GSE4922-GPL96	Disease free survival	249	0.88	0.010436	0.87	2.39 (1.23–4.65)
GSE7390	Relapse free survival	198	0.81	0.003653	0.71	2.03 (1.26–3.27)

Taken together, these results demonstrate that ATP5B is generally up-regulated in breast cancer tissue, suggesting that ATP5B may contribute to breast cancer progression, thus leading to unfavorable survival outcomes.

### Mutational Events Analysis

**FIGURE 5:**
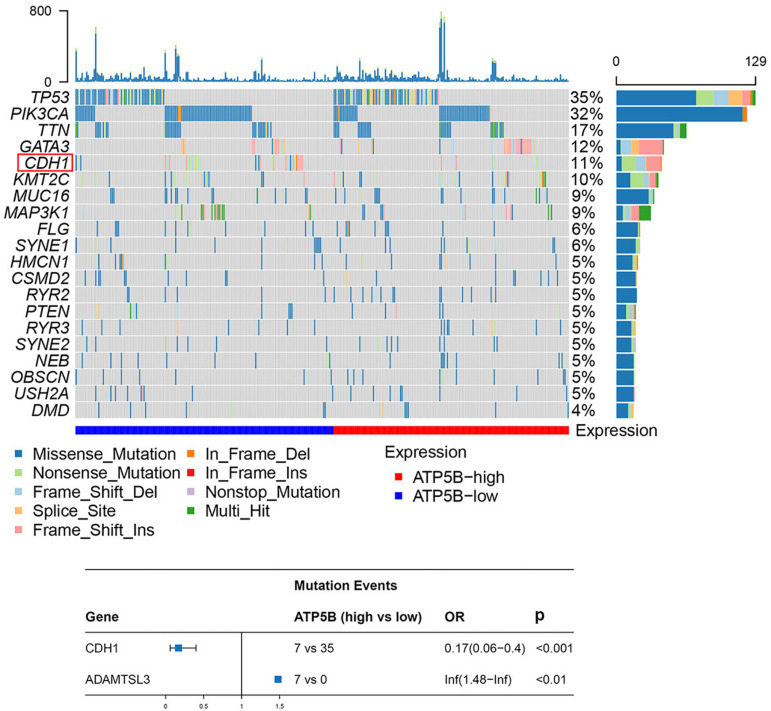
Mutation analysis of ATP5B. The top 20 mutated genes in the breast cancer subsets stratified by ATP5B expression. The red frame represents the mutation with a statistically significant difference (*p* < 0.05). At the bottom is a forest plot of significantly mutated genes. Consider only genes with at least three samples mutated in one of the cohort for analysis.

Previous genomic studies have revealed that genetic mutations play key roles in the development of breast cancer (Kwong et al., 2016; Lima et al., 2019). Thus, we evaluated the correlation of ATP5B expression with mutational profiles characterized in breast cancer. The results showed that the mutation frequency of two genes, CDH1 and ADAMTSL3, were statistically different between the ATP5B-high and ATP5B-low groups (Figure 5). ADAMTSL3, which has been proven to involve in cell proliferation in cancer (Jambaljav et al., 2018; Zhou et al., 2020), was only mutated in the ATP5B-high group. However, it is worth noting that CDH1, encoding the E-cadherin protein, was more frequently mutated in the ATP5B-low expression group. This finding supports a potential function related to the CDH1 and ADAMTSL3 mutations, which contributes to ATP5B overexpression and then affects the prognosis.

## Discussion

The molecular basis of improving long-term survival in breast cancer patients has not been fully studied. In this study, we analyzed the genetic characteristics of ATP5B in pan-cancer for the first time, including CNV, mRNA expression and DNA methylation as well as prognostic effects based on our dataset and public databases. In the breast cancer samples, ATP5B had higher mRNA and protein expression levels in cancer compared with the adjacent normal tissues, and higher ATP5B protein expression is closely associated with a worse clinical outcome.

ATP5B, an ATP synthase, is located on the cellular mitochondrial membrane, and it can be expressed ectopically on the surface of various types of cancer cells (Chi and Pizzo, 2006; von Ballmoos et al., 2008). ATP5B participates in crucial metabolic pathways in the body, such as in oxidative phosphorylation. Increased expression of ATP5B has been correlated with an unfavorable prognosis in certain cancers due to its promoting proliferation and metastasis of cancer cells (Xu and Li, 2016; Chung et al., 2020).

According to our pan-cancer analysis, results showed that both amplification and deletion of ATP5B are found in most cancer types, and the frequency of amplifications is greater than that of deletions. However, compared to normal tissues, ATP5B mRNA expression has distinct differences in various cancer types, suggesting that other elements may determine the expression of ATP5B, and its mechanism of function may be specific to different cancer types. Hence, we then analyzed the correlation between the mRNA expression level and the methylation level of ATP5B in pan-cancer, and identified the most relevant 6 methylation sites. These methylation sites had a significantly negative correlation with mRNA expression in most cancers, suggesting that methylation modification affected the expression level of ATP5B to some extent.

In breast cancer, we found that the expression level of ATP5B in diverse stages and subtypes of patients was higher than that in para-cancer normal tissues. Consistent with previous findings, ATP5B methylation levels were significantly down-regulated in patients with different stages and molecular subtypes of breast cancer relative to that in normal tissues. In addition, our data demonstrated that higher expression of ATP5B is significantly associated with a shorter OS in patients with breast cancer. We used both univariate and multivariate analyses to determine that ATP5B expression is an independent prognostic factor. At the same time, we verified the prognostic effect of ATP5B in three breast cancer data sets, and the results were consistent.

Through mutation analysis based on ATP5B mRNA expression, CDH1 was identified to have a higher mutation frequency in the ATP5B-low group. This result was surprising, as E-cadherin is a marker of epithelial cells and loss of it customarily has been considered to mark the beginning of epithelial-mesenchymal transformation, thus causing invasion and metastasis (Frixen et al., 1991). Interestingly, the latest studies have demonstrated that E-cadherin functions as an invasion suppressor and survival factor in breast invasive ductal carcinoma, which is the most common type of breast cancer (Dossus and Benusiglio, 2015; Padmanaban et al., 2019). GSEA analysis was also enriched in the E-cadherin nascent pathway in the ATP5B-high group. These results suggest that the expression of ATP5B is associated with the function of E-cadherin in breast cancer. Increased expression of ATP5B may cause invasion and metastasis through E-cadherin and its related pathways, leading to a worse prognosis. Further experiments are necessary to verify the mechanism of ATP5B in patients with breast cancer.

In summary, we demonstrated that mRNA and protein expression of ATP5B are both increased in breast cancer, and higher expression of ATP5B is closely associated with a worse prognosis. This study has highlighted the potential clinical value of ATP5B in predicting the outcomes of patients with breast cancer.

## Data Availability Statement

The original contributions presented in the study are included in the article/Supplementary Material, further inquiries can be directed to the corresponding author/s.

## Ethics Statement

The studies involving human participants were reviewed and approved by the Ethics Committee of Tianjin Medical University Cancer Institute and Hospital. The patients/participants provided their written informed consent to participate in this study.

## Author Contributions

ML, and LB conceived and designed the research. ML, YX, YZ, and YM performed the experiments. ML, ZS, JZ, and RL analyzed the data. ML wrote the original draft. LB edited and formed the final version. LB obtained funding and supervised the project. All authors have read and approved the final version of the manuscript.

## Conflict of Interest

The authors declare that the research was conducted in the absence of any commercial or financial relationships that could be construed as a potential conflict of interest.

## Footnotes

1 https://xenabrowser.net/

2 http://molonc.bccrc.ca/aparicio-lab/research/metabric/

3 http://www.prognoscan.org/

## Supplementary Material

Supplementary Table 1 CNV frequency of ATP5B and the correlation between ATP5B CNV and mRNA expression in pan-cancer.

Supplementary Table 2 The clinicopathological features of all patients in the TMA cohorts.
